# Association Between Ethnicity and Treatment Preferences in Patients with Irritable Bowel Syndrome

**DOI:** 10.5041/RMMJ.10542

**Published:** 2025-04-29

**Authors:** Vered Richter, Efrat Broide, Tzippora Shalem, Daniel L. Cohen, Tawfik Khoury, Atallah Mansour, Timna Naftali, Amir Mari

**Affiliations:** 1 The Gonczarowski Family Institute of Gastroenterology and Liver Disease, Shamir (Assaf Harofeh) Medical Center, Zerifin, Israel; 2 Faculty of Medicine, Tel Aviv University, Tel Aviv, Israel; 3 The Jecheskiel Sigi Gonczarowski Pediatric Gastroenterology Unit, Shamir (Assaf Harofeh) Medical Center, Zerifin, Israel; 4 Gastroenterology and Hepatology Institute, Nazareth Hospital EMMS, Nazareth, Israel; 5 The Azrieli Faculty of Medicine, Bar Ilan University, Israel; 6 Department of Gastroenterology, Meir Medical Center, Kfar Saba, Israel

**Keywords:** Arabs, ethnicity, irritable bowel syndrome (IBS), Jewish, patient preferences

## Abstract

**Background and Aims:**

Irritable bowel syndrome (IBS) poses a significant healthcare challenge, characterized by chronic gastrointestinal and extraintestinal symptoms impacting individuals’ well-being. Treatment preferences may vary among patients from different ethnic groups, such as Arab and Jewish Israelis, necessitating tailored approaches.

**Methods:**

A bilingual (Hebrew/Arabic) questionnaire assessing patients’ preferences regarding treatment goals was developed. It was administered online in Israeli IBS Facebook groups, as well as in two hospital gastroenterology clinics.

**Results:**

The study included 267 IBS patients (91 Arabs and 176 Jews). Demographic analysis revealed a higher proportion of females in both groups, with a significantly greater percentage among Jews compared to Arabs (84% versus 64.8%, respectively, *P*<0.001). The median age was 32 years for both Arabs and Jews (interquartile ranges of 26–42 and 24–62, respectively). Arabs exhibited higher rates of mixed-type IBS and constipation, while Jews had a higher prevalence of predominant diarrhea IBS. Arabs reported more bloating, higher rates of IBS-related comorbidities, and more medication usage. When asked to rate the importance of treatment goals, both populations preferred improvement in abdominal pain, bloating, and regular defecation, while assigning lower importance to improving difficulty in mental and/or physical aspects of intercourse, as well as arthralgia and myalgia. Arab patients assigned lower importance scores to various symptoms compared to their Jewish counterparts.

**Conclusion:**

This study highlights the impact of ethnicity on patients’ treatment goals. Understanding patients’ preferences will enable tailoring an individual approach to each IBS patient.

## INTRODUCTION

Irritable bowel syndrome (IBS) is a functional disorder of the gastrointestinal tract characterized by chronic abdominal pain and changes in bowel habits. These changes may include constipation, diarrhea, or a combination of both.^1^ It is a prevalent condition in the realm of healthcare surveillance, exerting a substantial financial burden on health systems.^2^,^3^ Those affected by IBS experience a significant decline in their quality of life, affecting their physical, psychological, and social well-being.^4^ Recently, a study on the epidemiology of all disorders of gut–brain interaction in Israel found that the overall prevalence of IBS was 3.2%, with a subtype distribution of 38.5% IBS with constipation, 30.8% IBS with diarrhea, 18.5% IBS-unspecified, and 12.3% IBS-mixed.^5^

Irritable bowel syndrome manifests with a variety of physical symptoms, encompassing abdominal pain, alterations in bowel habits, bloating, and extraintestinal disorders such as headaches, joint pains, sleep disturbances, and sexual dysfunction.^6^–^8^ This condition is often linked to other health issues, including fibromyalgia, gastroesophageal reflux disease (GERD), and psychiatric disorders.^8^,^6^ Individuals with IBS exhibit a three-fold increased likelihood of experiencing anxiety or depression compared to their healthy counterparts.^9^

Hence, it becomes imperative to extend the evaluation beyond clinical symptoms alone and scrutinize factors influencing patients’ functionality and overall lifestyle. Additionally, research indicates that sexual dysfunction contributes to a diminished quality of life in women with IBS.^10^

Very often, discordance between patient preferences and physician goals hamper treatment success and frustrate both sides. In the domain of gastrointestinal diseases, various questionnaires (including Patient-Reported Outcomes) concerning diverse therapeutic goals, encompassing clinical, mental, and social goals, and more, have been developed to bridge the communication gap between patients and clinicians. Presumably, patients from different backgrounds have different expectations from treatment outcomes. Previous IBS studies were performed separately in Jewish and Arab Israelis due to differences between the population groups.^11^–^13^

Our study endeavors to explore the divergent expectations regarding treatment among IBS patients within both Arab and Jewish populations. By doing so, we aim to heighten physicians’ awareness of the potential impact of ethnic background on treatment goals in managing this condition. The instrument utilized in our research has been meticulously tailored to accommodate the unique characteristics of IBS, thereby fostering a more comprehensive understanding of the syndrome within both communities.

## MATERIALS AND METHODS

### Study Population and Design

Patients with IBS, aged 18–75, were eligible for the study. The questionnaire was published on various Facebook groups concerning Israeli IBS patients and was available from January 2021 through December 2023. Patients self-reported that a physician had diagnosed them with IBS according to the Rome IV criteria. Additionally, a hard copy of the questionnaire was given to IBS patients who attended gastroenterology clinics at Nazareth Hospital EMMS and Shamir Medical Center and were diagnosed with IBS per the Rome IV criteria but had not previously completed the online questionnaire. Ethnicity (Arabs or Jews) was defined according to the Israeli Bureau of Statistics.

### Questionnaire

The questionnaire was developed by a team of highly experienced gastroenterologists, including both Jewish and Arab doctors, from Nazareth Hospital EMMS, Meir Medical Center, and Shamir Medical Center. It was formulated in Hebrew based on daily interactions with patients in routine clinical practice, then translated into Arabic by a bilingual expert to maintain the conceptual integrity of the questions. To ensure translation accuracy, the Arabic version underwent reverse translation by a different bilingual expert back into Hebrew.

The questionnaire was divided into two sections. The first section included demographic details, information about the type and duration of IBS, related diseases and symptoms, and current medical treatment. The second section consisted of 11 various symptoms related to IBS, with patients rating the importance they attach to the improvement of each symptom on a Likert scale of 1–10 (1 being least preferred and 10 being most preferred). Additionally, patients were requested to prioritize the top three improvements from treatment they considered most crucial. The complete questionnaire is provided as a supplement.

### Ethical Consideration

The analysis of data adhered to the principles outlined in the Declaration of Helsinki. The study received approval from the Institutional Review Board of Nazareth Hospital EMMS (Approval No. 32-21-EMMS) and Shamir Medical Center (Approval No. 0064-22 -ASF). Informed consent was waived by the Ethics Committee since the questionnaire was completed anonymously, and the act of responding to the questionnaire was considered implicit consent.

### Statistical Analyses

Categorical variables were presented as frequencies and percentages. The distribution of continuous variables was assessed through histograms and the Kolmogorov–Smirnov test. Normally distributed continuous variables were described using the median with interquartile range, or mean and standard deviation (SD). For comparisons of categorical variables, the chi-square test or Fisher’s exact test was employed, while independent Student’s *t*-test or the Mann–Whitney test was used for continuous and ordinal variables. A significance level of *P*<0.05 was adopted, and all statistical tests were two-sided.

## RESULTS

### Demographics of the Study Population

The research study included 267 participants with a diagnosis of IBS, comprising 91 individuals from the Arab population and 176 from the Jewish population. The demographic characteristics of patients were compared between Arabs and Jews, and the results are presented in Table 1. As expected, based on published data,^14^ both groups had a higher percentage of women, with more women among Jews compared to Arabs (84% versus 64.8%, respectively, *P*<0.001). The median age for both Arabs and Jews was 32 (interquartile ranges [IQR] 26–42 and 24–62, respectively). No statistically significant differences were observed in terms of comorbidities between the two groups nor for income and educational levels. Residency in a city was prevalent in both groups. However, statistically significant differences emerged between the groups, with 42.8% of Arabs living in a relationship compared to 57.1% of Jews (*P*=0.02), and 85.7% Arabs in employment compared to 60.2% of Jews (*P*<0.001) (see Table 1 for details).

**Table 1:** Baseline Characteristics of the Study Population.

Characteristic	Ethnicity		*P* Value

	Arabs (*n*=91)	Jews (*n*=176)	
Female, *n* (%)	59 (64.8)	147 (84)	**<0.001**

Age, median (IQR), years	32 (26–42)	32 (24–62)	0.22

Comorbidity*, *n* (%)	59 (64.81)	119 (67.6)	0.64
Ischemic heart disease	1 (1.1)	5 (2.8)	0.66
Hyperlipidemia	5 (5.5)	17 (9.7)	0.24
Diabetes mellitus	4 (4.4)	12 (6.8)	0.42
Hypertension	11 (12.1)	26 (14.8)	0.54
Fibromyalgia	11 (12.1)	16 (9.1)	0.44
Migraine	11 (12.1)	22 (12.5)	0.92

Education, *n* (%)			
12 years or less	34 (37.4)	72 (40.9)	0.57
More than 12 years	57 (62.6)	104 (59.1)	

Residency in a city, *n* (%)	72 (79.1)	147 (83.5)	0.37

In a relationship, *n* (%)	39 (42.8)	100 (57.1)	**0.02**

Working, *n* (%)	78 (85.7)	106 (60.2)	**<0.001**

Income, *n* (%)			
Below average	53 (58.2)	84 (56.3)	0.13
Average	15 (16.5)	32 (26.2)	
Above average	23 (25.3)	26 (17.4)	

Statistically significant results are indicated in **bold**, with a *P* value less than 0.05.

* Comorbidity included: ischemic heart disease, hyperlipidemia, diabetes mellitus, hypertension, fibromyalgia, migraine, sleep disorders, anxiety, and gastroesophageal reflux disease.

IQR, interquartile range.

### IBS Characteristics in Jewish and Arab Patients

In terms of the characteristics of IBS, significant differences were observed among the groups with respect to the type of IBS, the prevalence of IBS-related comorbidities, and medications usage. Notably, there was a statistically significant disparity between Arabs and Jews in the distribution of IBS types. Among Arabs, mixed-type IBS (52.7%) and predominant constipation IBS (28.6%) were more prevalent, whereas predominant diarrhea IBS was more common among Jews (39.2%, *P*<0.001). Arabs also exhibited a higher prevalence of abdominal bloating compared to Jews (96.7% versus 84.7%, respectively, *P*=0.003). Sleep disorders, anxiety, and GERD were all significantly more prevalent among Arabs, as indicated in Table 2.

**Table 2:** Patient IBS Characteristics and Treatment.

IBS Characteristic	Ethnicity		*P* Value
	Arabs (*n*=91)	Jews (*n*=176)	
IBS subtype			
Unclassified	7 (7.7)	31 (17.6)	**<0.001**
With predominant diarrhea	10 (11)	69 (39.2)	
With predominant constipation	26 (28.6)	29 (16.5)	
With mixed bowel habits	48 (52.7)	47 (26.7)	
Associated comorbidities/symptoms			
Bloating	88 (96.7)	149 (84.7)	**0.003**
Sleep disorders	32 (35.2)	34 (19.3)	**0.004**
Anxiety	28 (30.8)	31 (17.6)	**0.014**
Gastroesophageal reflux disease	36 (39.6)	40 (22.7)	**0.004**
Disease duration, median (IQR), years	10 (4–15)	6.5 (2.7–15)	**0.06**
Medication treatments			
Any medication	87 (95.6)	119 (67.6)	**<0.001**
Painkillers*	34 (37.4)	49 (27.8)	0.11
Antidepressant	41 (45.1)	37 (21)	**<0.001**
Sleep medication	30 (33)	15 (8.5)	**<0.001**
Cannabis	3 (3.3)	15 (8.5)	0.10
Probiotics	36 (39.6)	64 (36.4)	0.60
Rifaximin	5 (5.5)	7 (4.0)	0.57
Antidiarrheal	9 (9.9)	20 (11.4)	0.71
Antispasmodic	34 (37.4)	41 (23.3)	**0.01**
Laxative	28 (30.8)	11 (6.3)	**<0.001**
Pancreatic enzymes	2 (2.2)	2 (1.1)	0.60
Fiber supplement	53 (58.2)	16 (9.1)	**<0.001**
Non-medical treatment			
Emotional support	10 (11)	40 (22.7)	**0.02**
Homeopathic/alternative	77 (84.6)	50 (28.4)	**<0.001**
Psychiatric treatment	11 (12.1)	15 (8.5)	0.35
Does the treatment help?			
No	12 (13.2)	43 (29.1)	**0.01**
Partially	59 (64.8)	79 (53.4)	
Yes	20 (22)	26 (17.5)	

* Painkillers include non-steroidal anti-inflammatory drugs, opiates, dipyrone, paracetamol, and others.

IBS, irritable bowel syndrome; IQR, interquartile range.

Statistically significant results are indicated in BOLD, with a p-value less than 0.05.

Regarding treatment, Arabs showed a significantly higher prevalence of medication usage compared to Jews, particularly with respect to antidepressants, sleep medications, stool softeners, and antispasmodic medications. Additionally, other treatment modalities such as fiber supplements, dietary interventions, emotional approaches, and homeopathic/alternative methods were also more commonly employed by Arabs, as detailed in Table 2.

### Rating the Importance of Medical Treatment in Improving Various IBS Symptoms in the Study Cohort

**Figure 1:**
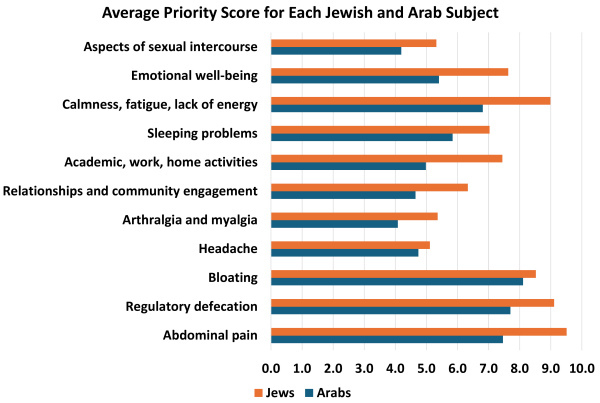
Average Priority Scores among Jewish and Arab Patients.

Arabs assigned significantly lower scores for medical treatment across all parameters, with the exception of the perceived importance of improving headaches (4.74 for Arabs compared to 5.11 for Jews, *P*=0.53). The disparities continued in the evaluation of specific symptoms, such as abdominal pain. Similar trends were observed in the assessment of regulatory defecation, enhancing daytime calmness, and addressing fatigue and lack of energy (Figure 1). The aspects that received lower ratings were the difficulty in mental and/or physical aspects of sexual intercourse, and arthralgia and myalgia. A comprehensive overview of these findings is illustrated in Figure 1.

**Figure 2:**
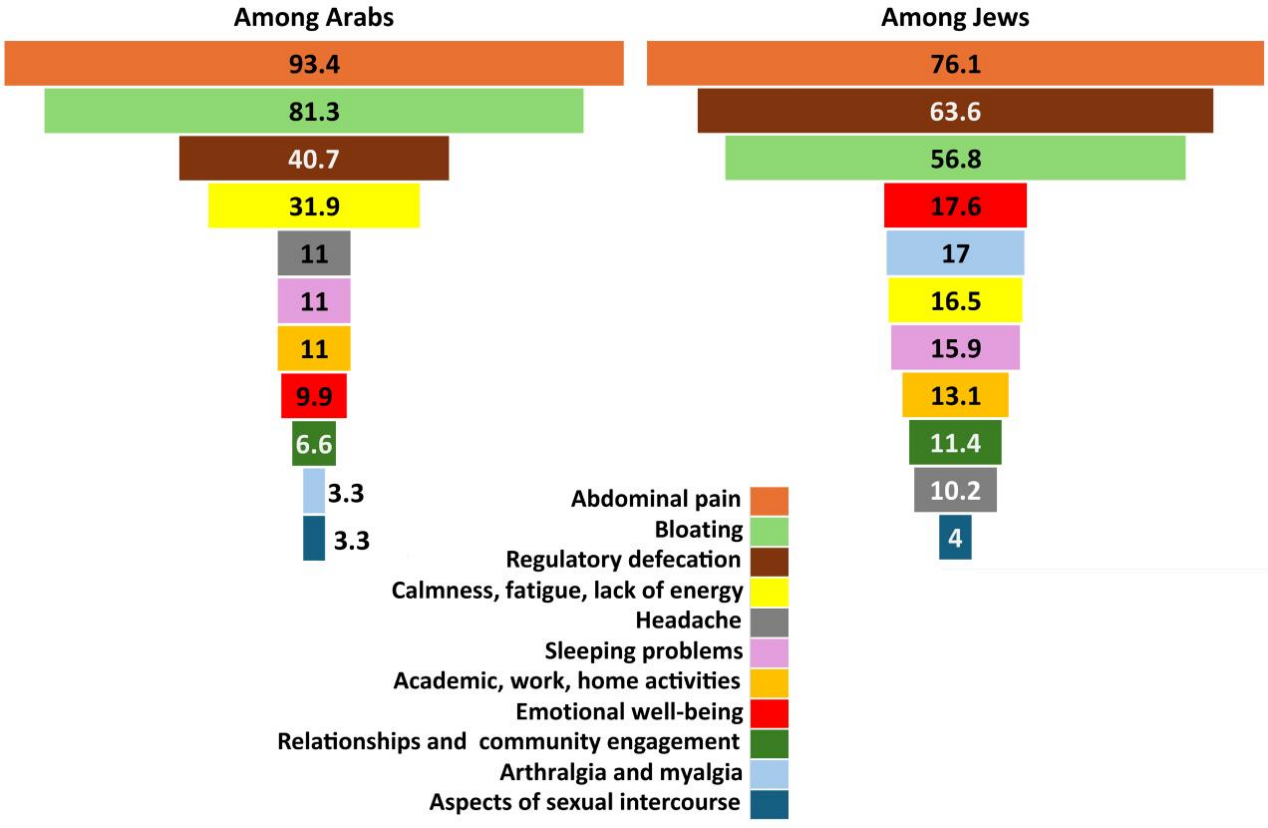
Percentage Distribution of Top Three Crucial Improvements Identified by Jewish and Arab Patients.

Among the mentioned symptoms, patients were asked to prioritize the top three improvements that they deemed most crucial. Figure 2 illustrates the percentage of patients selecting each symptom. Notably, the majority of both Arab (93.4%) and Jewish (76.1%) patients identified the improvement in abdominal pain as the most crucial. For Arabs, the second priority was reducing bloating (81.3%), while, for Jewish patients, it was regular defecation (63.6%). Conversely, the improvement of difficulty in mental and/or physical aspects of sexual intercourse ranked among the least important for both groups, with only 3.3% of Arabs and 4% of Jews selecting it among their top three priorities (*P*=0.78).

## DISCUSSION

This comparative study between Israeli Arabs and Jews with IBS shows overall similarities between the two groups regarding their demographic characteristics. However, while their demographics were quite similar, the disease phenotypes and, consequently, goals and preferences differed. Thus, the study provides a nuanced overview of the differences in IBS between the two communities.

Israeli Arabs (Muslims, Christians, and Druze) make up about 21% of the total population of Israel. Ethnicity was categorized based on the Israeli Central Bureau of Statistics classification into religious ethnic backgrounds of two main groups, Arabs and Jews.^14^ Several studies have compared these groups in different clinical settings,^15^–^19^ including studies that have shown genetic differences between them.^20^–^23^

Due to its recognized importance as a treatment goal, the measure of patients’ preferences and reported outcomes has been extensively studied in the last decade for various medical conditions including oncological, rheumatological, and gastrointestinal diseases.^24^–^26^ Notably, the influence of ethnicity and cultural backgrounds on patients’ preferences and goals of treatment is generally missed. A previous study by our group assessed the impact of ethnicity (Arabs and Jews) on patient preferences and treatment goals among an IBD cohort, using the IBD-disk 10-item questionnaire.^27^ The main findings included that symptom relief was the highest priority in both groups. The prioritization of symptoms in this current study, with both groups emphasizing the importance of alleviating abdominal pain, provides a clear directive for clinicians to focus on pain management as a central component of IBS treatment plans. Also, ethnicity, gender, and socioeconomic disparity impacted patients’ ranking priorities for the various treatment outcomes, findings that advocate for a more personalized approach in the clinical management of IBS.

One major finding of our study is the difference in disease phenotypes among patients from different ethnicities. Arab IBS patients had more constipation predominance and bloating, whereas Jewish IBS patients presented more frequently with diarrhea. This could be explained by various genetic, cultural, behavioral, nutritional, and probably geographical differences.^28^ Moreover, microbiome structural variances might exist in different ethnic populations and might provide more insights into the observed IBS phenotypes.^28^ Regional disparities in healthcare access and health literacy likely confound the observed differences in IBS treatment preferences and outcomes between Arab and Jewish Israelis, highlighting the need for culturally sensitive and accessible care. Knowledge of this variation in IBS characteristics between Arabs and Jews may help personalize treatment regimens to address these specific symptoms more effectively.

Healthcare utilization by means of pharmacological therapies, nutritional consultation, psychological, and alternative (homeopathic) medicine approaches was also more prevalent amongst Arab patients. This could be related to the higher prevalence of IBS-related comorbidities among Arab patients in our study. One interesting point is the extensive use of alternative and homeopathic therapies to control IBS symptoms. We assume that cultural “herbal” medicine in the Arab community constitutes a major part of alternative/homeopathic therapies.^29^,^30^ These findings may underscore the necessity for comprehensive management plans that integrate pharmacological and non-pharmacological treatments, including dietary interventions and emotional support.

Principally, we observed that patients of Jewish ethnicity attached higher rank to most questionnaire items than did the Arab ethnic group. This difference is most likely due to differences in lifestyles and cultural influences. This finding is in line with our previous study of the IBD-disk tool.^28^ Patients from both populations ranked abdominal pain as the top priority symptom to target, whereas Arab patients ranked bloating and constipation in the second and third priorities compared to diarrhea and bloating symptoms among the Jewish IBS population. These findings are in parallel with previous reports where IBS patients from different ethnicities have shown various preferences and treatment targets. Almario et al. conducted a large-scale nationwide survey shedding light on the influence of cultural and demographic factors on IBS. They found significant variations in IBS prevalence and symptom severity across racial and ethnic groups. Abdominal pain was more severe among blacks and Hispanics with IBS with diarrhea.^31^ In our study, while both Arabs and Jews identified the improvement in abdominal pain as the most crucial, Arab participants assigned significantly higher importance scores. Also, in our study, Arabs had more associated comorbidities such as bloating. A study conducted by Silvernale et al. retrospectively matched white IBS patients to Hispanic, Asian, and black patients. They found that white patients underwent fewer endoscopic procedures and more in-clinic consultations compared to the other racial groups.^32^ A more recent study by Boyd et al. evaluated disparities in IBS management among white patients compared to blacks/African Americans and found that black patients were less likely to be referred to a dietician for consultation and were prescribed more medications for constipation.^33^ In a comprehensive review paper, Sasegbon et al. thoroughly discussed the impact of race on IBS management approach, as well as patient-related factors affecting clinician decisions.^34^ The lower ratings of the importance of medical treatment in improving IBS symptoms among Arabs may call for culturally sensitive communication strategies to ensure that patients fully understand and appreciate the benefits of various treatment options.

Our study has some limitations. Many of the study variables were defined based on self-reporting, and therefore reporting bias on some variables might exist. In addition, we relied on a self-reported diagnosis of IBS. However, physicians routinely use the Rome IV questionnaire in clinical settings to diagnose patients with IBS. We also included data from participants diagnosed at two gastroenterology clinics, where Rome IV criteria were formally applied. Since selection bias was a potential concern given the online questionnaire, distributing the questionnaire in gastroenterology clinics helped mitigate this issue. Despite these limitations, the study has several strong points. It used a questionnaire developed by senior gastroenterologists, was delivered in two languages, and was distributed digitally—all of which enabled greater accessibility.

## CONCLUSIONS

To the best of our knowledge, this is the first study to address the impact of ethnicity on IBS patients’ preferences and treatment outcomes. Our study findings reinforce the idea that ethnicity and culture impact treatment preferences in IBS and that this may direct a tailored and personalized approach to an IBS patient’s care.

## Supplementary Information



## Abbreviations

GERD: gastroesophageal reflux disease
IBS: irritable bowel syndrome
IQR: interquartile range
SD: standard deviation
