# Supplementary material for: Association Between Ethnicity and Treatment Preferences in Patients with Irritable Bowel Syndrome
Source: Rambam Maimonides Med J. 2025 Apr 29;16(2):e0007. doi: 10.5041/RMMJ.10542 (PMC12053809; doi:10.5041/RMMJ.10542)
Supplement: Supplementary file 1 [file rmmj-16-2-e0007_Supplement.pdf]

SUPPLEMENTARY MATERIAL

This appendix has been provided by the authors for the benefit of readers

Supplement to Association Between Ethnicity and Treatment Preferences in Patients with Irritable Bowel Syndrome

Richter V, Broide E, Shalem T, Cohen DL, Khoury T, Mansour A, Naftali T, Mari A. Association Between Ethnicity and Treatment Preferences in Patients with Irritable Bowel Syndrome. Rambam Maimonides Med J 2025;16 (2):e0007. doi:10.5041/RMMJ.10542

ENGLISH TRANSLATION OF THE STUDY QUESTIONNAIRE

Dear patients, we present here a short questionnaire aimed at understanding which symptoms related to irritable bowel syndrome are most important for you to improve or resolve through treatment. There are no right or wrong answers. We would appreciate it if you answer the questionnaire to the best of your ability.

1. What is your gender?
   - Male
   - Female
2. What is your age? _______
3. What is your ethnic origin?
   - Ashkenazi Jew
   - Sephardi Jew
   - Mixed Jewish
   - Ethiopian Jew
   - Former Soviet Union
   - Arab

4. What is your religion?
    - o Jewish
    - o Muslim
    - o Christian
    - o Other
5. What is your religious observance?
    - o Secular
    - o Traditional
    - o Religious
    - o Ultra-Orthodox
6. How many years of schooling do you have? ________
7. What is the highest level of educational institution that you graduated from?
    - o Elementary school
    - o High school
    - o Yeshiva (religious school)
    - o College (Bachelor's degree)
    - o University (Bachelor's degree)
    - o University (Master's degree)
    - o University (Doctorate)
8. Do you live in a:
    - o City
    - o Community settlement
    - o Moshav (cooperative agricultural settlement)
    - o Kibbutz (collective agricultural settlement)
    - o Other
9. What is your marital status?
    - o Single
    - o Married
    - o In a relationship but not married
    - o Widowed
    - o Divorced

10. Do you work?
    - o Yes
    - o No

    If yes, what is your average number of working hours per week? _____
11. The average salary in Israel, as of October 2019, was 10,501 NIS per month. Your income level compared to the national average is:
    - o Much higher
    - o Higher
    - o Average
    - o Below average
    - o Much below average
12. Apart from irritable bowel syndrome, do you suffer from any of the following conditions?
    - o Hypertension
    - o Diabetes
    - o Chronic lung disease
    - o Heart disease
    - o High cholesterol
    - o Cancer
    - o Migraines
    - o Fibromyalgia
    - o Depression and/or anxiety
    - o Sleep disorders
    - o Gastroesophageal reflux disease (GERD)
    - o Other chronic illnesses
    - o I do not suffer from any underlying illnesses
13. Do you take the following medications?
    - o Antidepressants/anxiolytics
    - o Sleeping pills
    - o Painkillers (non-steroidal anti-inflammatory drugs, opioids, etc.)
    - o Cannabis
    - o None of the above
    - o Don’t know/not sure

14. Is your irritable bowel syndrome predominantly characterized by:
    - Diarrhea
    - Constipation
    - Mixed
    - Undefined
15. Do you suffer from bloating?
    - Yes/No
16. Do you suffer from excessive gas?
    - Yes/No
17. How many years have you been diagnosed with irritable bowel syndrome? _______
18. What medications do you use for irritable bowel syndrome?
    - Dietary fiber
    - Probiotics
    - Non-absorbable antibiotics
    - Laxatives
    - Antidiarrheals (loperamide)
    - Antispasmodics (e.g., Colotal, Spasmalgin)
    - Antidepressants/anxiolytics
    - Natural and homeopathic products
19. Are you undergoing any of the following non-medication treatments?
    - Nutritional therapy
    - Emotional/psychological/group therapy
    - Psychiatric treatment
    - Homeopathic treatment
20. Does the treatment help you?
    - Yes, very much
    - Yes, it helps
    - It helps partially
    - Not at all

Below is a list of symptoms related to your irritable bowel syndrome. Please rate how important it is to you that medical treatment successfully improves each symptom, on a scale of 1 (not at all important to me that the treatment improves this) to 10 (very important to me that the treatment improves this)

1. It is important to me that the treatment improves my abdominal pain
2. It is important to me that the treatment improves the changes in my bowel habits
3. It is important to me that the treatment improves the bloating
4. It is important to me that the treatment improves my headaches
5. It is important to me that the treatment improves my joint/muscle pain
6. It is important to me that the treatment improves difficulties in personal relationships and/or difficulties participating in the community
7. It is important to me that the treatment improves difficulties with academic studies and/or difficulties with work or activities at home
8. It is important to me that the treatment improves sleep difficulties such as difficulty falling asleep, frequent awakenings during the night, or waking up too early in the morning
9. It is important to me that the treatment improves feelings of calmness and freshness during the day, and reduces feelings of fatigue and lack of energy
10. It is important to me that the treatment improves sadness, low mood or depression, and/or worry and anxiety
11. It is important to me that the treatment improves difficulties with the mental and/or physical aspects of sexual relations

From all the aforementioned symptoms, please select the 3 symptoms that are most important for you to improve with treatment
